# The Warburg Effect Reinterpreted 100 yr on: A First-Principles Stoichiometric Analysis and Interpretation from the Perspective of ATP Metabolism in Cancer Cells

**DOI:** 10.1093/function/zqae008

**Published:** 2024-02-21

**Authors:** Sunil Nath, Rudi Balling

**Affiliations:** Department of Biochemical Engineering and Biotechnology, Indian Institute of Technology Delhi, Hauz Khas, New Delhi 110016, India; Institute of Molecular Psychiatry, Rheinische-Friedrichs-Wilhelm Universität Bonn, D‒53127 Bonn, Germany; Institute of Molecular Psychiatry, Rheinische-Friedrichs-Wilhelm Universität Bonn, D‒53127 Bonn, Germany

**Keywords:** aerobic glycolysis and the Warburg Effect, cancer, malignancy, and heterogeneity, metabolic coupling and symbiosis, mathematical model, oxidative phosphorylation (OXPHOS) and F_0_F_1_-ATP synthase, lactate and lactic acid, biomass yield coefficients based on ATP, Nath’s two-ion theory of energy coupling and torsional mechanism of ATP synthesis, Nath’s unified theory of ATP synthesis/hydrolysis, Warburg-Nath ratio; Nath-Warburg number, NaWa; metabolic regulation based on ATP demand and supply, stoichiometry and available electron balance

## Abstract

The Warburg Effect is a longstanding enigma in cancer biology. Despite the passage of 100 yr since its discovery, and the accumulation of a vast body of research on the subject, no convincing biochemical explanation has been given for the original observations of aerobic glycolysis in cancer cell metabolism. Here, we have worked out a first-principles quantitative analysis of the problem from the principles of stoichiometry and available electron balance. The results have been interpreted using Nath’s unified theory of energy coupling and adenosine triphosphate (ATP) synthesis, and the original data of Warburg and colleagues have been analyzed from this new perspective. Use of the biomass yield based on ATP per unit substrate consumed, ${{Y}_{X/S}}\ ATP$, or the Nath-Warburg number, NaWa has been shown to excellently model the original data on the Warburg Effect with very small standard deviation values, and without employing additional fitted or adjustable parameters. Based on the results of the quantitative analysis, a novel conservative mechanism of synthesis, utilization, and recycling of ATP and other key metabolites (eg, lactate) is proposed. The mechanism offers fresh insights into metabolic symbiosis and coupling within and/or among proliferating cells. The fundamental understanding gained using our approach should help in catalyzing the development of more efficient metabolism-targeting anticancer drugs.

## Graphical Abstract

**Graphical Abstract:**
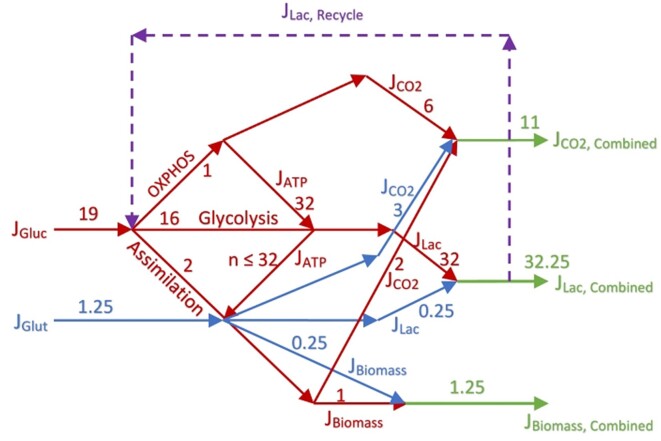

## Introduction

The year 2024 marks the 100th anniversary of discovery of the Warburg Effect. Influential research in the 1920s by Warburg and colleagues and by Cori and Cori demonstrated that cancer cells consume glucose and excrete lactate at high rates in the presence of oxygen.^1–4^ In their landmark paper, Warburg and colleagues reported quantitative measurements of respiration and lactic acid production in a variety of normal and cancerous tissues using manometric techniques.^1^ This phenomenon of aerobic glycolysis, named the Warburg Effect by Racker,^5^ has been subsequently confirmed in a variety of tumor types.^6–9^

The Warburg Effect is so very reliable an effect in most tumor types that it has been routinely employed for medical diagnosis of tumors using positron emission tomography (PET) imaging with a radioisotope-labeled glucose tracer, 2-deoxy-2-^18^F-fluoro-β-D-glucose (^18^F-FDG) that identifies areas of high glucose uptake or metabolism in the human body. Positron emission tomography scans reveal increased glucose uptake due to overexpression of glucose transporters (GLUT) in tumors.^10–12^  ^18^F-FDG is transported into cells by GLUT and phosphorylated to ^18^F-FDG-6-phosphate by the action of hexokinase (HK). Owing to its highly polar nature, ^18^F-FDG-6-phosphate is trapped within cells and is not further metabolized through the glycolytic pathway. Hence, tumors above a critical size are labeled strongly using ^18^F-FDG, and the technique is commonly used to identify the presence of solid tumors and monitor the efficacy of various pharmacological and drug treatments.

Despite the above medical developments and applications of the Warburg Effect in cancer diagnosis, no fully convincing biochemical explanation has been put forth for the observations on aerobic glycolysis by cancer cells. The observations contradict the known facts on the inefficiency and low yield of glycolysis to make the universal biological energy carrier adenosine triphosphate (ATP) compared to ATP synthesis by the oxidative phosphorylation (OXPHOS) pathway,^13–15^ though some rationalizing arguments and mathematical models have been proposed.^16,17^ Warburg himself advanced the theory that mitochondria are dysfunctional, and he postulated that such respiratory injury and impairment is a universal metabolic characteristic of carcinogenesis.^18,19^

Advances in technology have, however, revealed that cancer cells possess active and functional mitochondria.^20–23^ While some studies have implicated increased glycolysis as being the major cause of malignancy,^24^ other works^25^ have suggested that the efficiency of mitochondrial energy conversion^14,26,27^ is the key metabolic factor. In fact, no explanation for the difference in the extent or grade of malignancy among cancer cell types has been provided.^28,29^ Thus, contrary to Warburg’s theory, cancer cells have increased mitochondrial activity in a large group of human cancers.^30–32^ Hence, there exists a complex and dynamic interplay between OXPHOS and glycolysis in different tumor types, and within tumor subpopulations. These have been discussed in the large literature on intercellular metabolic coupling and the Reverse Warburg Effect,^23^,^32–40^ first proposed by Martinez-Outschoorn and coworkers.^33,34^ Intracellular coupling and regulation of ATP^41–44^ provides other possibilities. In an important review and consolidation of data on cancer cell energetics, Zu and Guppy showed that on average, only 17% of the ATP in various cancer types is derived from glycolysis—with the remaining ATP being obtained from OXPHOS—and the range of glycolysis varying from 0.31% for fibrosarcoma to 64% for hepatoma.^45^

A central role for lactate OXPHOS in cancer cells has been described in detail. The hypothesis on lactate metabolism was first made by Gladden^46^ and by Brooks on the presence of lactate shuttles in cancer cells,^47^ to our knowledge. Experimental verification was first obtained in 2009,^39^ and further experimental details were uncovered recently.^48^^‒^^51^ A number of reviews re-evaluating the role of lactate in cancer metabolism and signal transduction have since been published.^52–59^ Recently, Brooks made a plea to consider the lactate anion, as opposed to H^+^, on cancer cell metabolism.^53^ Similar proposals to consider the central role of the succinate anion have been made in mitochondrial cell energetics in health and disease since the turn of the century by Nath^60^ and reviewed subsequently in great detail by researchers,^61–64^ and experimental support provided for the crucial role of the dicarboxylic acid anion in energy coupling and vesicle acidification.^65^

A large number of major articles have been published due to a resurgence of interest on the Warburg Effect.^23,34,39,40^,^53–55^,^57–59^,^66–78^ Almost all of these articles are reviews that survey a part of the vast literature. Some have pointed out difficulties with previous explanations of the effect.^40,66,67^ Others have highlighted the need for further discussion to better understand the effect.^70^ Many deal with interesting facets of the problem. For example, the fact that the Warburg Effect is a key feature of cell growth rather than cell division has been emphasized.^71^ The need for conducting experiments at physiological conditions of pO_2_ ($\sim50\ $mm Hg) and glucose ($\sim5\ $m m) in cancer cells, as opposed to high pO_2_ (160 mm Hg) and/or elevated glucose concentrations of 25 or 11 m m typically employed in cancer cell culture studies, which can lead to glucose inhibition of respiration, have been highlighted.^23,74,78^ There are also contradictory features. For example, several reviews stress the switching of metabolism from OXPHOS to glycolysis in cancer cells,^58,69^ while others claim no such switch exists.^55^ These claims shall be adjudicated by independent quantitative analysis in the present work. Moreover, original insights that can shed new light on the Warburg Effect are, on the whole, lacking in the review articles.

Here, we have attempted to take a fresh look at the problem from the perspective of ATP metabolism in cancer cells. We have applied Nath’s two-ion theory of energy coupling^79–82^ and torsional mechanism of ATP synthesis^60,83,84^ to cancer cells along with the basic principles of mass balance and available electron balance in cell metabolism. This is shown to provide quantitative new insights into the problem. In Germany, for the centenary year, the authors have also read and dissected the papers written by Warburg in German, and have analyzed the original data based on the Nath’s unified ATP theory.

More than 50 000 papers with the keywords “Warburg Effect” and “ATP” have been published in the cancer literature, with a surge in the number of papers since the new millennium until today. However, this is the first report using the above-mentioned novel approach to the problem, to the best of our knowledge.

The conservation of mass is a general law that can be used to determine the quantities of various species entering or leaving a cell, or any bioprocess for that matter.^85–87^ When dealing with mass balances in the presence of chemical reaction(s), it is required to know the stoichiometry of conversion/reaction. In particular, when growth or proliferation occurs, as in cancer cells, the cells must be accounted for by representation in the chemical reaction equations as a product of the reaction(s). The theory is described in the “Theory” section, and the methods in the “Methods” section. Various results obtained are given in “Estimation of Yield Coefficients,” “Calculations of Fractions,” “Available Electron Balances,” “Calculation of the Approximate Contributions of Glucose Carbons and Glutamine Carbons to Biomass, Lactate, and CO_2_ in a Proliferating Cell,” “The Warburg Effect,” “Analysis of Warburg’s Original Data on Aerobic Glycolysis By Rat and Human Carcinoma,” and “Analysis of Warburg’s Subsequent Data Collected on Aerobic Glycolysis” sections. The results are extensively discussed in the “Discussion” section. Such metabolic stoichiometric analysis, along with the available electron balance (see the “Methods” section) and use of ATP theory is shown here to have interesting biological applications to cancer cells and to offer novel insights into the origin, phenomenology, and understanding of the Warburg effect. The section “Concluding Remarks and Biological Implications” formulates and lays down the conclusions and biological implications arising from this study.

## Theory

**Figure 1.**
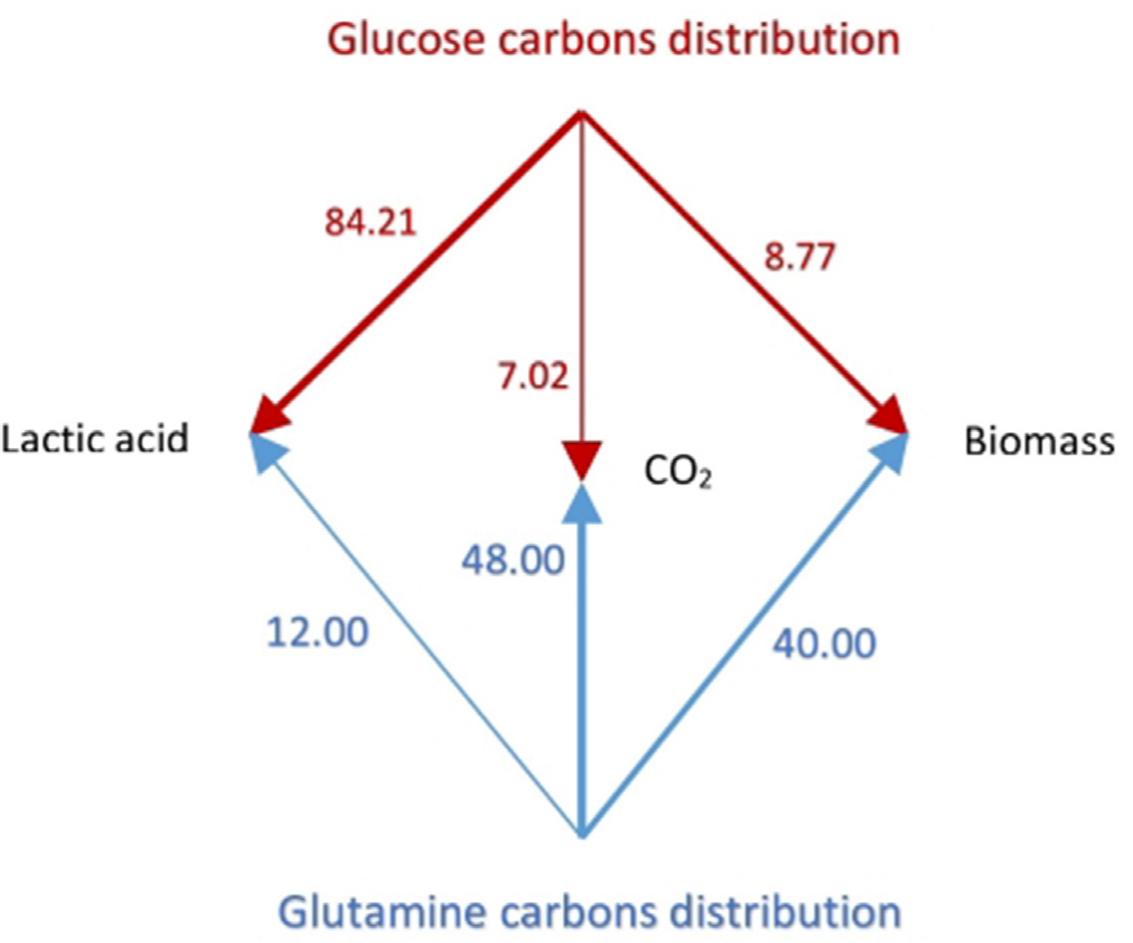
Distribution of glucose and glutamine carbons (wt. %) in biomass, lactic acid, and CO_2_ in a proliferating cell.

Despite the occurrence of hundreds of intracellular chemical reactions and their complexity, all cells, including cancer cells obey the law of conservation of mass. The elemental balances for carbon, hydrogen, nitrogen, and oxygen atoms need to be satisfied during cell growth and proliferation, that is, the atoms of these elements are either incorporated into new cell mass or into products that are exported out, or further utilized in other cellular processes. Figure 1 represents a macroscopic view of cell metabolism. It does not include the detailed structure of the cellular system, but considers the main metabolites that are exchanged with the surroundings. The overall macroscopic balance shown in Figure 1, despite its simplicity, provides a powerful approach and offers important information for thermodynamic analysis of the biosystem, especially if the intracellular cycles of ATP and key metabolites are included, as shown in this work.

In order to formulate a stoichiometric and energetic balance for the type of process represented by Figure 1, an empirical formula for dry biomass needs to be used that is based on typical cell compositions. Different cells under various cell culture conditions and substrates utilized can, however, be represented by an “average” stoichiometric formula. With $\sim$10% ash added to account for elements not included in the average empirical formula for cell biomass, a fairly accurate overall description of the cellular system is obtained.^85–87^ We would like to emphasize that the principal results of this work using the biomass yield based on ATP consumed, ${{Y}_{X/S}}\ ATP$ given by eqs (32)‒(37), (46)‒(48), (52)‒(55), (58), and (59) are based on calculations using Warburg’s data on oxygen consumed and lactic acid produced [1] and the regularity observed in the biomass yield of ATP for various cell types, and are rather insensitive to the stoichiometry of the empirical formula for biomass used in the calculations. That said, this study would gain from experimental determination of a more exact empirical formula for biomass of specific types of cancer cells, for example, by a $C,H,\ N$ elemental analysis. It also ought to be emphasized that the calculations can be easily repeated or re-done if an alternative representation or metric is employed, or if different stoichiometric equations are used. The equations should be considered as a minimal representation of cancer cell metabolism that capture the essential carbon and nitrogen assimilation into biomass and satisfactorily model Warburg’s original data.^1,4^

The aforementioned has stressed the fact that the present work presents a *parsimonious* theory of cancer metabolism that, in essence, adequately describes Warburg’s data.^1,4^ For instance, possible reliance of some cancers additionally on fatty acid oxidation, or leading also to alanine production as a by-product, can be included in the equations to describe other features of cancer metabolism. The flux of carbon/nitrogen from/into these additional substrates/products need to be reliably known to effect these modifications. The concept of zero waste and complete recycle of intermediates in steady-state cellular function and the approach of conservation of available electrons used to formulate and analyze the stoichiometric balance equations are described in the “Methods” section.

### Overall Balance Equations

The overall stoichiometric equations can now be written for the average cancer cell based on the following principal assumptions:

1. Lactic acid is the primary product of proliferating cells; other products, for example, alanine consume only 1%-2% of substrate carbon.^88^
2. Glucose and glutamine substrates are each metabolized separately into biomass;
3. Ammonia is the nitrogen source, which can be derived from glutamine metabolism;
4. The total yield of ATP from glucose is given by Nath’s torsional mechanism of ATP synthesis^13,14,60^ and regulation based on consideration of both demand and supply sides^41,42^ (Table 1).

**Table 1.** Yield of ATP from glucose

Reaction or Value	Number Based on Nath’s Unified Theory of ATP Synthesis/Hydrolysis and Regulation^41^^,42^
Glycolysis	2
2 NADH from glycolysis	7.5
8 NADH from TCA cycle	30
2 FADH from TCA cycle	4.5
2 GTP from 2 succinyl CoA	2
Total maximum ATP per glucose	46
Total maximum ATP per glucose from OXPHOS	7.5 + 30 + 4.5 = 42
Actual or effective number of ATP per glucose from OXPHOS (η = 66.67%)	28
Total actual number of ATP per glucose	2 + 28 + 2 = 32
Calculated actual or operative P/O ratio in OXPHOS	$\frac{{10}}{{8/3}} \times 0.667 = 2.5$
Experimental mean P/O ratio in OXPHOS on NADH substrates	2.55
Total [O] atoms consumed	12
Total actual ATP per [O] in the cell	32/12 = 2.67

The metabolic reaction equations are


(1) \begin{eqnarray*}
{{C}_6}{{H}_{12}}{{O}_6} + 6\ {{O}_2} \to 6\ C{{O}_2} + 6\ {{H}_2}O
\end{eqnarray*}

for the complete oxidation of glucose;


(2) \begin{eqnarray*}
16\ {{C}_6}{{H}_{12}}{{O}_6} \to 32\ {{C}_3}{{H}_6}{{O}_3}
\end{eqnarray*}

representing metabolism by the glycolytic pathway (substrate-level phosphorylation), assuming that the entire pool of ATP is accessible for use by the proliferating cell;


(3) \begin{eqnarray*}
2\ {{C}_6}{{H}_{12}}{{O}_6} + 2.5\ {{O}_2} + 2\ N{{H}_3} \to {{C}_{10}}{{H}_{16}}{{N}_2}{{O}_6} + 2\ C{{O}_2} + 7\ {{H}_2}O
\end{eqnarray*}

showing the partial oxidation of glucose, for the average biomass composition given by the first term on the right-hand side of eqn (3);


(4) \begin{eqnarray*}
&&1.25\ {{C}_5}{{H}_{10}}{{N}_2}{{O}_3} + 2.5\ {{O}_2} \to 0.25\ {{C}_{10}}{{H}_{16}}{{N}_2}{{O}_6}\\
&&\qquad + 0.25\ {{C}_3}{{H}_6}{{O}_3} + 3\ C{{O}_2} + 2\ N{{H}_3} + 0.5\ {{H}_2}O
\end{eqnarray*}

for glutamine oxidation.

Adding eqns (1), (2), and (3), we obtain an overall reaction [eqn (5)] for glucose oxidation:


(5) \begin{eqnarray*}
&&19{\mathrm{\ }}{{C}_6}{{H}_{12}}{{O}_6} + 8.5\ {{O}_2} + 2\ N{{H}_3} \to {{C}_{10}}{{H}_{16}}{{N}_2}{{O}_6} + 32\ {{C}_3}{{H}_6}{{O}_3}\\
&&\qquad + 8\ C{{O}_2} + 13\ {{H}_2}O.
\end{eqnarray*}

The overall balanced reaction for combined glucose and glutamine metabolism by the cancer cell can therefore be written by summation of eqn (4) and eqn (5) as eqn (6):


(6) \begin{eqnarray*}
&&19{\mathrm{\ }}{{C}_6}{{H}_{12}}{{O}_6}\ + \ 1.25\ {{C}_5}{{H}_{10}}{{N}_2}{{O}_3} + 11\ {{O}_2} \to 1.25\ {{C}_{10}}{{H}_{16}}{{N}_2}{{O}_6}\\
&&\qquad + 32.25\ {{C}_3}{{H}_6}{{O}_3} + 11\ C{{O}_2} + 13.5\ {{H}_2}O.
\end{eqnarray*}

## Methods

The principles of mass and energy balance in biochemical reactions have been well described in several books and monographs,^85–87^ and is summarized in the “Theory” section. In addition, we have used the concepts of zero accumulation of intermediates in the steady state, and the conservation of available electrons.

When writing the balanced metabolic reactions, we have ensured the complete cycling of ATP and NH_3_. Thus, whatever amount of these intermediates are produced are utilized by other processes. Thus, there is no net accumulation of these metabolites during steady-state operation. This greatly limited the number of possibilities available to us in deriving the balance equations given in the “Theory” section.

The available electron balance that allowed us to quantify heat and other losses requires further explanation. Available electrons refer to the number of electrons available for transfer to oxygen, biomass, product, etc. during oxidation of a substrate. The number of available electrons in an organic substrate or product is calculated from the valence of the various elements contained in the species. The reference state for cell growth is taken to be the same as that of the nitrogen source in the medium, for example, ammonia. The available electron balance is often written in terms of the degree of reduction, *γ*, which is defined as the number of equivalents of available electrons in an amount of organic material containing 1 g atom carbon. On this basis, the degrees of reduction of substrate, biomass, and product can be readily calculated, as shown in the “Available electron balances” section. For example, for a substrate ${{C}_w}{{H}_x}{{O}_y}{{N}_z}$, the number of available electrons is $( {4w + x - 2y - 3z} )$. The degree of reduction of the substrate, ${{\gamma }_S}$ is then given by $( {4w + x - 2y - 3z} )/w$.

The electrons that are available for transfer to oxygen are conserved during cell growth and metabolism. This conservation principle arises because the masses of each element is conserved in a balanced reaction equation. On this basis, eqns (25)‒(27) can be written for glucose, and similarly for other substrates such as glutamine. These mass and available electron conservation equations have been used to derive various relationships and to calculate the metabolic yield coefficients in “Estimation of Yield Coefficients,” “Calculations of Fractions,” “Available Electron Balances,” “Calculation of the Approximate Contributions of Glucose Carbons and Glutamine Carbons to Biomass, Lactate, and CO_2_ in a Proliferating Cell,” “The Warburg Effect,” “Analysis of Warburg’s Original Data on Aerobic Glycolysis By Rat and Human Carcinoma,” and “Analysis of Warburg’s Subsequent Data Collected on Aerobic Glycolysis” sections.

## Results

### Estimation of Yield Coefficients

Let *S* stand for substrate (glucose or glutamine), *P* for the principal product (lactate), *X* for biomass, and let *Y* represent the corresponding yield coefficient (g g^‒1^). With this notation, the following results are obtained for the theoretical values of the various yield coefficients calculated stoichiometrically based on eqn (6):


(7) \begin{eqnarray*}
{{Y}_{X/S}}\ \textit{Glucose} &=& \frac{{{{C}_{10}}{{H}_{16}}{{N}_2}{{O}_6}}}{{19\ {{C}_6}{{H}_{12}}{{O}_6}}}\\
&&\quad = \frac{{12 \times 10 + 16 \times 1 + 2 \times 14 + 6 \times 16}}{{19\left( {12 \times 6 + 12 \times 1 + 6 \times 16} \right)}} = \frac{{260}}{{19 \times 180}}\\
&&\quad = \frac{{260}}{{3420}} = 0.076\ g{{g}^{ - 1}},
\end{eqnarray*}

and similarly,


(8) \begin{eqnarray*}
{{Y}_{X/S}}\ \textit{Glutamine} = \frac{{0.25 \times 260}}{{1.25 \times 146}} = \frac{{65}}{{182.5}} = 0.356\ g{{g}^{ - 1}},
\end{eqnarray*}

(9) \begin{eqnarray*}
{{Y}_{X/S}}\ \textit{Overall} = \frac{{1.25 \times 260}}{{19 \times 180 + 1.25 \times 146}} &=& \frac{{325}}{{3420 + 182.5}} = \frac{{325}}{{3602.5}}\\
&&\quad = 0.090\ g{{g}^{ - 1}},
\end{eqnarray*}

(10) \begin{eqnarray*}
{{Y}_{P/S}}\ \left( {\textit{Lactic}\ \textit{acid}/Glucose} \right) = \frac{{32 \times 90}}{{19 \times 180}} = \frac{{16}}{{19}} = 0.842\ g{{g}^{ - 1}},
\end{eqnarray*}

(11) \begin{eqnarray*}
{{Y}_{P/S}}\ \left( {\textit{Lactic}\ \textit{acid}/Glutamine} \right) = \frac{{0.25 \times 90}}{{1.25 \times 146}} = \frac{{18}}{{146}} = 0.123\ g{{g}^{ - 1}},\\
\end{eqnarray*}

(12) \begin{eqnarray*}
{{Y}_{P/S}}\ \left( {\textit{Lactic}\ \textit{acid}/Overall} \right) &=& \frac{{32.25 \times 90}}{{19 \times 180 + 1.25 \times 146}}\\
&&\quad = \frac{{2902.5}}{{3602.5}} = 0.806\ g{{g}^{ - 1}},
\end{eqnarray*}

(13) \begin{eqnarray*}
{{Y}_{ATP}}\,\,\left( {on\,\, {Glucose}} \right) = \frac{{260}}{{32}} = 8.125\,\, g\,\,dry\,\,{cell}\,\,{({mol\ ATP})}^{ - 1},
\end{eqnarray*}

(14) \begin{eqnarray*}
&&{{Y}_{ATP}}\ \left( {on\ \textit{Glucose} + \textit{Glutamine}} \right) = \frac{{1.25 \times 260}}{{32}}\\
&&\quad = \frac{{325}}{{32}} = 10.156\,\,{g\,\,dry\,\,cell\,\,({mol\,\,ATP})^{ - 1}},
\end{eqnarray*}

(15) \begin{eqnarray*}
{{Y}_{C{{O}_2}/Glucose}} = \frac{{8 \times 44}}{{19 \times 180}} = \frac{{352}}{{3420}} = 0.103\ g{{g}^{ - 1}},
\end{eqnarray*}

(16) \begin{eqnarray*}
{{Y}_{C{{O}_2}/Glutamine}} = \frac{{3 \times 44}}{{1.25 \times 146}} = \frac{{132}}{{182.5}} = 0.723\ g{{g}^{ - 1}},
\end{eqnarray*}

(17) \begin{eqnarray*}
{{Y}_{C{{O}_2}/\left( {\textit{Glucose} + \textit{Glutamine}} \right)}} &=& \frac{{11 \times 44}}{{19 \times 180 + 1.25 \times 146}} = \frac{{484}}{{3602.5}}\\
&&\quad = 0.134\ g{{g}^{ - 1}}.
\end{eqnarray*}

Equations (7)‒(17) give a more or less complete set of cell yield coefficients based on stoichiometric principles.

### Calculation of Fractions

We can also calculate the fraction of biomass, product, etc. produced by each of the 2 substrates, glucose and glutamine, and other fractional quantities as follows:


(18) \begin{eqnarray*}
&&{\rm Fraction\,\, of\,\, cell\,\, biomass\,\, contributed\,\, by\,\, glucose} = \frac{{1}}{{1.25}} = 0.80,\\
&&{\rm Fraction\,\,of\,\,cell\,\,biomass\,\,contributed\,\,by\,\,glutamine} = \frac{{0.25}}{{1.25}} = 0.20,\\
\end{eqnarray*}

(19) \begin{eqnarray*}
&&{\rm Fraction\,\,of\,\, product\,\, lactic\,\, acid\,\, contributed\,\, by\,\, glucose}\\
&&\qquad = \frac{{32}}{{32.25}} = 0.992,\\
&&{\rm Fraction\,\,of\,\,product\,\,lactic\,\,acid\,\,contributed\,\,by\,\,glutamine}\\
&&\qquad = \frac{{0.25}}{{32.25}} = 0.008,
\end{eqnarray*}

(20) \begin{eqnarray*}
&&{\rm Fraction\,\, of}\,\, {CO_2}\,\,{\rm contributed\,\, by\,\, glucose} = \frac{8}{{11}} = 0.727,\\
&&{\rm Fraction\,\,of}\,\,{CO_2}\,\,{\rm contributed\,\, by\,\, glutamine} = \frac{3}{{11}} = 0.273,\\
\end{eqnarray*}

(21) \begin{eqnarray*}
&&{\rm Fraction\,\,of\,\,total\,\,glucose\,\,uptake\,\,utilized\,\,for\,\,complete\,\,respiration}\\
&&\qquad = \frac{1}{{19}} = 0.053,\\
&&{\rm Fraction\,\,of\,\,glucose\,\,utilized\,\,for\,\,incomplete\,\,respiration} = \frac{2}{{19}}\\
&&\qquad = 0.105,\\
&&{\rm Fraction\,\,of\,\,glucose\,\,used\,\,for\,\,lactic\,\,acid\,\,formation} = \frac{{16}}{{19}}\\
&&\qquad = 0.842.
\end{eqnarray*}

Based on eqn (5),


(22) \begin{eqnarray*}
&&{\rm Fraction\,\, of\,\, glucose\,\, carbon\,\, assimilated\,\, into\,\, biomass}\\
&&\qquad = \frac{{10}}{{19 \times 6}} = 0.088,\\
&&{\rm Fraction\,\, of\,\, glucose\,\, carbon\,\, incorporated\,\, into\,\, product}\\
&&\qquad = \frac{{32 \times 3}}{{19 \times 6}} = 0.842,\\
&&\qquad {\rm Fraction\,\,of\,\,glucose\,\,carbon\,\,in\,\,{{CO}_2}} = \frac{8}{{19 \times 6}} = 0.070.
\end{eqnarray*}

Similarly, based on eqn (4),


(23) \begin{eqnarray*}
&&{\rm Fraction\,\,of\,\,glutamine\,\,carbon\,\,assimilated\,\,into\,\,biomass}\\
&&\qquad = \frac{{0.25 \times 10}}{{1.25 \times 5}} = 0.40,\\
&&{\rm Fraction\,\,of\,\,glutamine\,\,carbon\,\,incorporated\,\,into\,\,product}\\
&&\qquad = \frac{{0.25 \times 3}}{{1.25 \times 5}} = 0.12,\\
&&{\rm Fraction\,\,of\,\, glutamine\,\, carbon\,\, in}\,\,{CO_2} = \frac{3}{{6.25}} = 0.48.
\end{eqnarray*}

For the total (combined) substrate, we have


(24) \begin{eqnarray*}
&&{\rm Fraction\,\, of\,\, (glucose + glutamine)\,\, carbon\,\, in\,\,biomass}\\
&&\qquad = \frac{{1.25 \times 10}}{{19 \times 6 + 1.25 \times 5}} = 0.104,\\
&&{\rm Fraction\,\, of\,\, (glucose + glutamine)\,\, carbon\,\, in\,\, product}\\
&&\qquad = \frac{{32.25 \times 3}}{{19 \times 6 + 1.25 \times 5}} = 0.805,\\
&&{\mathrm{Fraction\ of\ }}\left( {{\mathrm{glucose\ }} + {\mathrm{\ glutamine}}} \right){\mathrm{\ carbon\ in\ }}C{{O}_2}\\
&&\qquad = \frac{{11}}{{19 \times 6 + 1.25 \times 5}} = 0.091.
\end{eqnarray*}

### Available Electron Balances

We can carry out an available electron balance as described in the “Methods” section based on the overall equation for glucose metabolism [eqn (5)] and glutamine metabolism [eqn (4)]. If $\gamma $ represents the degree of reduction, and subscripts $S,\ P,\ B$ stand for substrate, product, and biomass, respectively, and $\varepsilon $ represents fractions, we have the following balance,^86^


(25) \begin{eqnarray*}
{{\varepsilon }_B} + {{\varepsilon }_P} + {{\varepsilon }_{\textit{Losses}}} = 1.
\end{eqnarray*}

For glucose as substrate,


(26) \begin{eqnarray*}
{{\gamma }_S} = 4;\ {{\gamma }_P} = 4;\ {{\gamma }_B} = \frac{{10 \times 4 + 1 \times 16 - 2 \times 3 - 2 \times 6}}{{10}} = 3.8.
\end{eqnarray*}

Applying the available electron balance to substrate glucose, we have


(27) \begin{eqnarray*}
\frac{{10}}{{19 \times 6}} \times \frac{{3.8}}{4} + \frac{{32 \times 3}}{{19 \times 6}} \times \frac{4}{4} + \frac{{8.5}}{{19 \times 6}} \times \frac{4}{4} = \frac{{9.5}}{{114}} + \frac{{96}}{{114}} + \frac{{8.5}}{{114}} = 1.\\
\end{eqnarray*}

Thus,


(28) \begin{eqnarray*}
&&{\rm Fraction\,\, of\,\, available\,\, electrons\,\, of\,\, glucose\,\, assimilated\,\, into\,\, biomass}\\
&&\qquad = 0.083,\\
&&{\rm Fraction\,\, of\,\, available\,\, electrons\,\, of\,\, glucose\,\, incorporated\,\, into\,\, lactate}\\
&&\qquad = 0.842,\\
&&{\rm Fraction\,\, of\,\, losses,\,\, for\,\, example,\,\, as\,\, heat} = 0.075.
\end{eqnarray*}

With respect to substrate glutamine, we have


(29) \begin{eqnarray*}
{{\gamma }_S} = 3.6;\ {{\gamma }_P} = 4;\ {{\gamma }_B} = 3.8,
\end{eqnarray*}

(30) \begin{eqnarray*}
&&{\rm Available\,\, {e^-}\,\, fraction\,\, of\,\, glutamine\,\, in\,\, biomass} = 0.422,\\
&&{\rm Available\,\, {e^-}\,\, fraction\,\, of\,\, glutamine\,\, in\,\, lactate} = 0.133,\\
&&{\rm Available\,\,{e^-}\,\,fraction\,\,of\,\,glutamine\,\,in\,\,oxygen}\,\, ({\rm losses}) = 0.444.\\
\end{eqnarray*}

Equations (7)‒(30) give us the various yield coefficients and fractions based on analysis of the stoichiometric eqns (1)‒(6).

### Calculation of the Approximate Contributions of Glucose Carbons and Glutamine Carbons to Biomass, Lactate, and CO_2_ in a Proliferating Cell

Based on the results of the “Available Electron Balances” section, and the ideal case where 32 ATP molecules are used per glucose for biosynthesis and product formation, we estimated the % distributions of glucose as well as glutamine carbons in biomass, lactic acid, and carbon dioxide. The results of these calculations based on eqns (22) and (23) are plotted in Figure 1.

**Figure 2.**
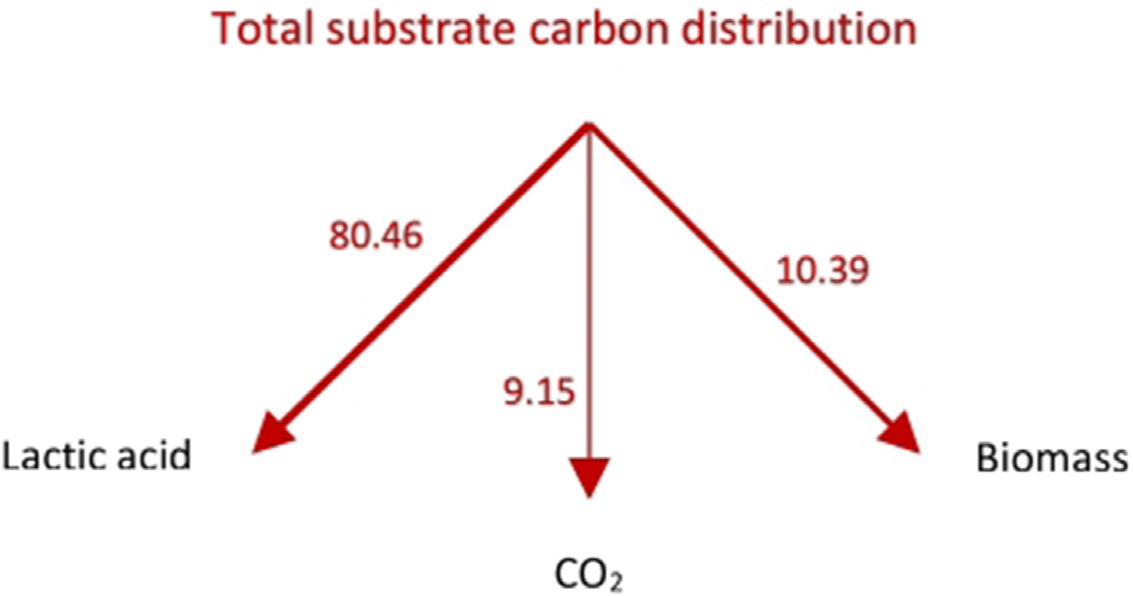
Distribution of total substrate carbon, that is, glucose and glutamine (wt. %) in biomass, lactic acid, and CO_2_ in a proliferating cell.

We also calculated the relative contributions of the total substrate carbon (glucose + glutamine) to biomass, lactic acid, and CO_2_ since we know (“Estimation of Yield Coefficients,” “Calculations of Fractions,” and “Available Electron Balances” sections) the fraction of carbon atoms derived from glucose as well as glutamine [see eqn (24)]. These results are shown in Figure 2.

**Figure 3.**
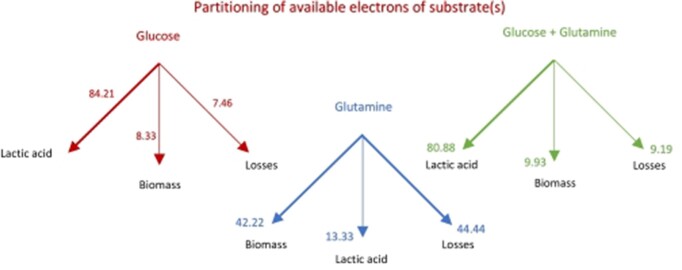
Distribution of available electrons (%) of glucose, glutamine, and combined (glucose + glutamine) substrates.

From the available electron balances made in the “Available Electron Balances” section, we quantified the partitioning of available e^‒^ of substrate(s) using the results of eqns (28) and (30). The results of this calculation are depicted in Figure 3.

### The Warburg Effect

**Figure 4.**
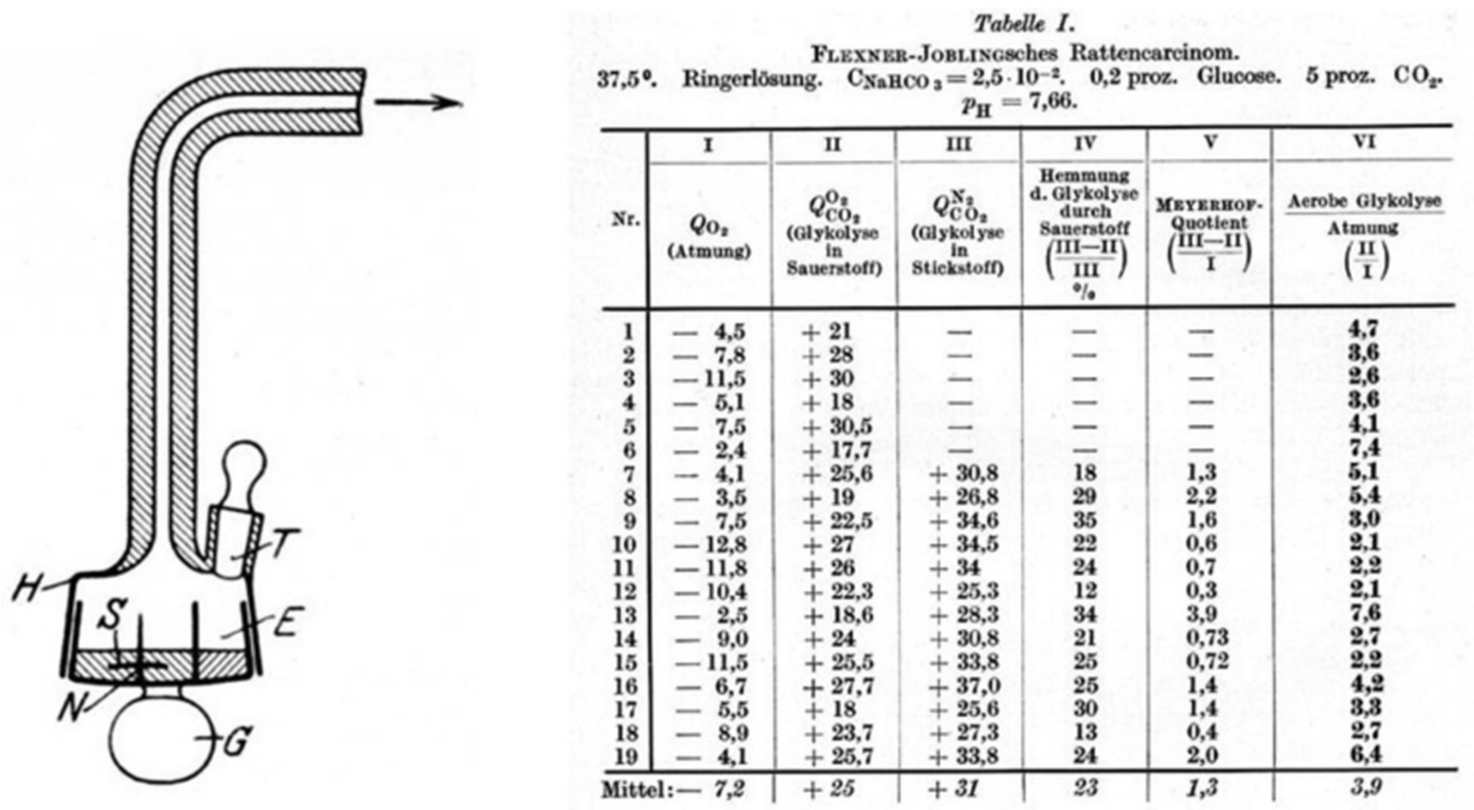
Apparatus used by Warburg and colleagues to measure aerobic glycolysis by rat and human carcinoma tissue (left). Representative data using the reaction vessel is shown in the table (right), which is taken from the original paper published 100 yr ago.^1^

Warburg employed manometric methods (Figure 4) to measure oxygen consumption in thin cancer tissue slices metabolizing glucose (see the “Overall Balance Equations” section). He also used the manometric technique to measure carbon dioxide liberation using bicarbonate buffers. As seen from eqn (31), 1 mole of CO_2_ emitted also produces 1 mole of lactate.


(31) \begin{eqnarray*}
\ {{C}_3}{{H}_6}{{O}_3} + HCO_3^ - \to {{C}_3}{{H}_5}{{O}_3}^ - + \ {{H}_2}O + C{{O}_2}
\end{eqnarray*}

Warburg and coworkers discovered the following:^1,3,4^

1. The Flexner-Jobling rat liver carcinoma slices have the same oxygen uptake as normal liver tissue, within error;
2. The cancer rat liver tissue slices generate lactic acid even in the presence of oxygen;
3. Glucose uptake by the Flexner-Jobling rat liver tissue carcinoma slices is elevated $\sim10 - 15$ times compared to that in normal cells.

Similar results were found by Warburg and colleagues for human carcinoma slices.^1^ They concluded that normal tissues cease lactic acid production in the presence of oxygen. However, cancerous tissues continue to process glucose and produce lactic acid even in the presence of O_2_ (“aerobic” glycolysis or the Warburg Effect), and inhibit/prevent the entry of pyruvate into the TCA cycle. They estimated from their measurements that the amount of lactic acid produced by cancer cells is approximately a couple orders of magnitude higher than that produced by normal cells.^1^

### Analysis of Warburg’s Original Data on Aerobic Glycolysis By Rat and Human Carcinoma

A stoichiometric and yield coefficient analysis of the mean values in the original data of Warburg and coworkers on the Flexner-Jobling rat carcinoma^1^ will now be illustrated based on the see the “Overall Balance Equations” and “Estimation of Yield Coefficients,” “Calculations of Fractions,” “Available Electron Balances,” and “Calculation of the Approximate Contributions of Glucose Carbons and Glutamine Carbons to Biomass, Lactate, and CO_2_ in a Proliferating Cell” sections.

In 1 h, per mg dry tissue the Warburg data showed,^1^


(32) \begin{eqnarray*}
&&{\rm Mean}\,\,{O}_2\,\,{\rm consumed}\\
&&\quad = 7.2\ m{{m}^3};{\rm the\,\, number\,\, of\,\, moles\,\, of}\,\,{O}_2\,\,{\rm consumed}\\
&&\quad = \frac{{1 \times 7.2 \times {{{10}}^{ - 3}}}}{{82.05 \times 310.5}} = 0.28\ \mu mol,
\end{eqnarray*}

(33) \begin{eqnarray*}
&&{\mathrm{Mean\ lactic\ acid\ produced\ }}\\
&&\quad = 25\ m{{m}^3};{\mathrm{\ the\ number\ of\ moles\ of\ lactic\ acid\ produced\ }}\\
&&\quad = \frac{{1 \times 25 \times {{{10}}^{ - 3}}}}{{82.05 \times 310.5}} = 0.98\ \mu mol.{\mathrm{\ }}
\end{eqnarray*}

Taking a basis of 1 h, per kg of dry tissue, we have, for the mean,


(34) \begin{eqnarray*}
{O}_2\,\,{\rm consumed} = 0.28\ mol,
\end{eqnarray*}

(35) \begin{eqnarray*}
{\mathrm{Lactic\ acid\ produced\ }} = 0.98\ mol,
\end{eqnarray*}

(36) \begin{eqnarray*}
{\mathrm{Glucose\ consumed\ for\ lactic\ acid\ production\ }}\\ = \frac{{0.98}}{2} = 0.49\ mol,
\end{eqnarray*}

(37) \begin{eqnarray*}
{\mathrm{Moles\ ATP\ produced}} = {\mathrm{\ moles\ lactic\ acid\ }} = 0.98\ mol,
\end{eqnarray*}

(38) \begin{eqnarray*}
&&{\mathrm{Glucose\ consumed\ for\ complete\ oxidation\ }}\left( {{\mathrm{OXPHOS}}} \right){\mathrm{\ }}\\
&&\qquad = \frac{{0.28}}{{8.5}} = 0.033\ mol,
\end{eqnarray*}

(39) \begin{eqnarray*}
&&{\mathrm{Glucose\ consumed\ for\ incomplete\ oxidation}}\\
&&\qquad = 2 \times 0.03294 = 0.066\ mol,
\end{eqnarray*}

(40) \begin{eqnarray*}
{\mathrm{Glucose\ consumed\ for\ complete\ }} &+& {\mathrm{\ incomplete\ oxidation\ }}\\
&&= 0.033 + 0.066 = 0.099\ mol,\\
\end{eqnarray*}

(41) \begin{eqnarray*}
&&{\mathrm{Total\ glucose\ consumed\ }}\\
&&\qquad \left( {{\mathrm{for\ oxidation\ }} + {\mathrm{\ lactate\ formation}}/{\mathrm{glycolysis}}} \right){\mathrm{\ }}\\
&&\qquad = 0.099 + 0.49 = 0.589\ mol,
\end{eqnarray*}

(42) \begin{eqnarray*}
{\mathrm{Mass\ of\ total\ glucose\ consumed\ }} = 0.589 \times 180 = 105.99\ g.
\end{eqnarray*}

Therefore, the Warburg-Nath ratio is equal to


(43) \begin{eqnarray*}
\frac{{\textit{Glucose}\ \textit{consumed}\ for\ \textit{glycolysis}}}{{\textit{Glucose}\ \textit{consumed}\ for\ \textit{OXPHOS}}} = \frac{{0.49}}{{0.03294}} = 14.875.
\end{eqnarray*}

We can perform a check of the above calculations. For example, using eqn (43) we obtain,


(44) \begin{eqnarray*}
&&{\mathrm{Moles\ ATP\ produced\ per\ mol\ glucose\ consumed\ by\ OXPHOS}}\\
&&\qquad = 2 \times 14.875 = 29.75\ mol,
\end{eqnarray*}

(45) \begin{eqnarray*}
&&{\mathrm{Therefore}},{\mathrm{\ moles\ of\ ATP\ produced\ per\ h\ per\ kg\ dry\ tissue\ }}\\
&&\qquad = 29.75 \times 0.033 = 0.98\ mol,
\end{eqnarray*}

which agrees with the estimate from material balance [eqn (37)].


(46) \begin{eqnarray*}
{\mathrm{Since\ }}{{Y}_{ATP}} \cong 10.5\ g\ dry\ \textit{cells}\ {{\left( {mol\ ATP} \right)}^{ - 1}}
\end{eqnarray*}

can be regarded as a constancy for bacterial cells,^86,89^ and as a constancy or a very good approximation for mammalian cells,^90^ we calculate that


(47) \begin{eqnarray*}
{\mathrm{Mass\ of\ biomass\ produced\ }} = 0.98 \times 10.5 = 10.29\ g.
\end{eqnarray*}

Hence, using eqns (42) and (47), we obtain the biomass yield based on ATP per unit substrate consumed, ${{Y}_{X/S}}\ ATP$ , the Nath-Warburg number


(48) \begin{eqnarray*}
NaWa = {{Y}_{X/S}}\ ATP = \frac{{10.29}}{{105.99}} = 0.097\ g{{g}^{ - 1}},
\end{eqnarray*}

(49) \begin{eqnarray*}
&&{\mathrm{The\ total\ glucose\ consumed\ per\ mol\ biomass\ produced\ }} = 1\\
&&\qquad + 14.875 + 2 = 17.875\ mol.
\end{eqnarray*}

By eqns (3) and (5),


(50) \begin{eqnarray*}
{\rm The\ molecular\ weight\ of\ biomass}\ = 260.
\end{eqnarray*}

Hence, using eqns (5) and (49), we obtain the actual biomass yield based on substrate glucose consumed ${{Y}_{X/S}}\ \textit{Glucose}$ as


(51) \begin{eqnarray*}
{{Y}_{X/S}}\ \textit{Glucose} = \frac{{260}}{{17.875 \times 180}} = 0.081\ g{{g}^{ - 1}}.
\end{eqnarray*}

The above value of actual biomass yield based on glucose consumed [eqn (51)] can be compared with the values of biomass yield based on ATP consumed ${{Y}_{X/S}}\ ATP$ [eqn (48)], and the theoretical biomass yield based on glucose obtained by stoichiometric calculation [eqn (7)].

**Figure 5.**
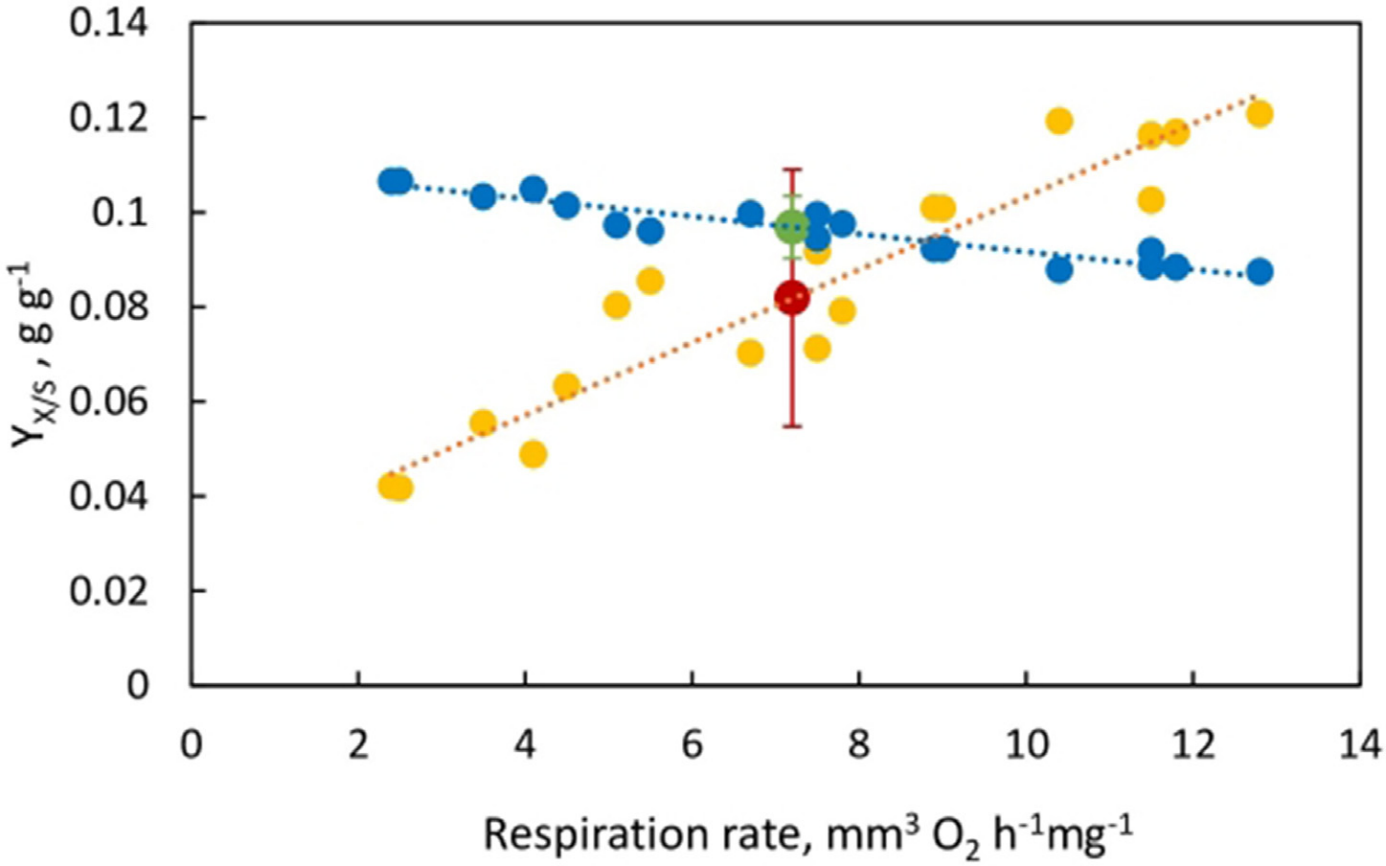
Biomass yield based on ATP consumed, ${{Y}_{X/S}}\ ATP$, filled blue circles (●) and theoretical biomass yield based on glucose, filled yellow circles (●) as a function of oxygen uptake rate for Warburg’s data on Flexner-Jobling rat carcinoma samples ($n = 19$).^1^  $\ \textit{Mean} \pm SD = 0.097 \pm 0.007$ based on $NaWa = {{Y}_{X/S}}\ ATP$: filled green circle (●). $Mean \pm SD = 0.082 \pm 0.027$ based on ${{Y}_{X/S}}\ \textit{Glucose}$: filled red circle (●).

The above representative calculations have been made for Warburg’s mean values of measurements on rat carcinoma.^1^ These calculations have been repeated for Warburg’s $n = 19$ rat tissue samples and the results are shown in Figure 5. The experimental results and their variation with O_2_ consumption rates have been shown for calculations based on both the ATP consumed as well as the actual substrate glucose consumed, and the mean $\pm $ SD have been reported (Figure 5).

**Figure 6.**
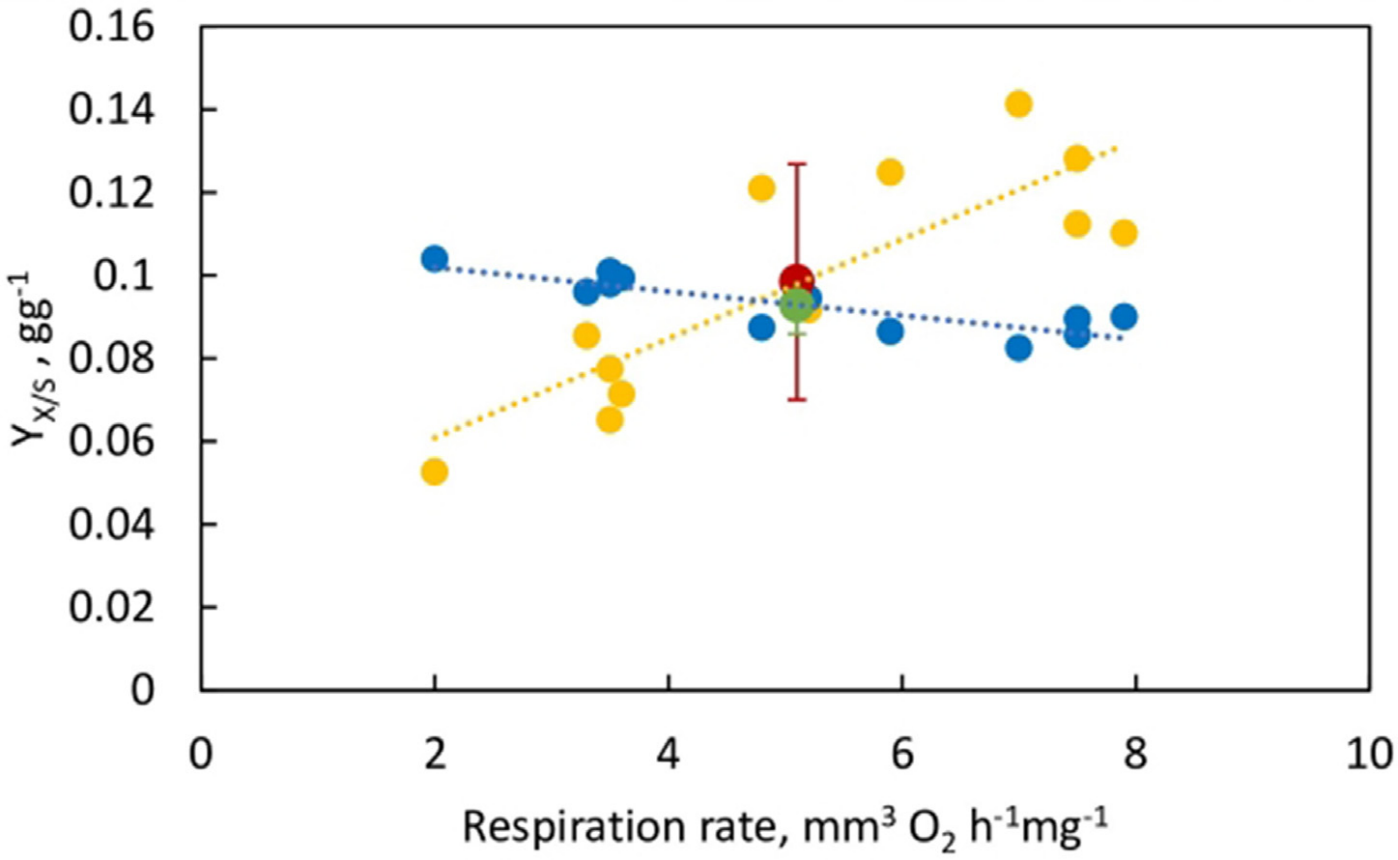
Biomass yield based on ATP consumed, ${{Y}_{X/S}}\ ATP$, filled blue circles (●) and theoretical biomass yield based on glucose, filled yellow circles (●) as a function of respiration rate for Warburg’s data on human carcinoma samples ($n = 12$).^1^  $\ \textit{Mean} \pm SD = 0.093 \pm 0.007$ based on $NaWa = {{Y}_{X/S}}\ ATP$: filled green circle (●). $Mean \pm SD = 0.098 \pm 0.028$ based on ${{Y}_{X/S}}\ \textit{Glucose}$: filled red circle (●).

Similar results on the yield coefficients as a function of the oxygen consumption rates measured on the human carcinoma tissues slices are shown in Figure 6.

It should be clearly understood that the calculated values of the biomass yield coefficients based on ATP and glucose shown in Figures 5 and 6 are based on Warburg’s original experimental data on respiration rates and lactic acid production in cancerous tissue^1^ and therefore include the presence of *heterogeneous* metabolism in cancer cells. Equations (48) and (51) illustrate a representative/model calculation of the coefficients for the *mean* value of respiration rates and lactic acid production rates reported by Warburg for his $n = 19$ tissue samples of the Flexner-Jobling rat carcinoma [see Table 1 of ref. [1] and Figure 4 of this work]. The model calculation was repeated for the 19 samples and 12 samples of the rat and human carcinoma, respectively, and used to generate the spread of calculated values of the yield coefficients in Figures 5 and 6.

Warburg and his colleagues showed amazing foresight in making the right measurements a century ago.^1,3,4^ The only statistical aspect that these authors missed was that they reported the results of single measurements for each data point (Figure 4). In the absence of repeated measurements, the uncertainty in the slope of the straight lines in Figures 5 and 6 cannot be determined with 100% confidence. We were therefore forced to assume that the measurements of Warburg and colleagues in their papers^1,3,4^ are exact. However, since their actual measurement uncertainties are not known, the slope uncertainty in Figures 5 and 6 may be an underestimate.

### Analysis of Warburg’s Subsequent Data Collected on Aerobic Glycolysis

Warburg and colleagues made further aerobic glycolysis measurements on Flexner-Jobling rat carcinoma and Jensen rat sarcoma.^3,4^ They found that, per 100 mL blood, the rat tumor removes 70 mg glucose and releases 46 mg lactic acid. Thus, using a basis of 100 L blood along with our earlier stoichiometric analysis (see “Estimation of Yield Coefficients,” “Calculations of Fractions,” “Available Electron Balances,” “Calculation of the Approximate Contributions of Glucose Carbons and Glutamine Carbons to Biomass, Lactate, and CO_2_ in a Proliferating Cell,” “The Warburg Effect,” and “Analysis of Warburg’s Original Data on Aerobic Glycolysis By Rat and Human Carcinoma” sections),


(52) \begin{eqnarray*}
{\mathrm{Total\ substrate\ glucose\ uptake\ }} = \frac{{70}}{{180}} = 0.39\ mol,
\end{eqnarray*}

(53) \begin{eqnarray*}
{\mathrm{Lactic\ acid\ produced\ }} = \frac{{46}}{{90}} = 0.51\ mol,
\end{eqnarray*}

(54) \begin{eqnarray*}
&&{\mathrm{Moles\ glucose\ required\ to\ produce\ lactic\ acid\ }}\\
&&\qquad = \frac{{0.51}}{2} = 0.255\ mol,
\end{eqnarray*}

(55) \begin{eqnarray*}
&&{\mathrm{Moles\ of\ ATP\ produced\ and\ used\ for\ biomass\ production}}\\
&&\qquad = 0.51\ mol.
\end{eqnarray*}

If $x = $ moles of glucose used for complete oxidation by the OXPHOS pathway, then the following relationship holds,


(56) \begin{eqnarray*}
0.39 - 3x = 0.255.
\end{eqnarray*}

(57) \begin{eqnarray*}
{\mathrm{Hence,\ }}x = 0.045\ mol\ {\mathrm{glucose\ inducted\ into\ OXPHOS}}.
\end{eqnarray*}

Using the constancy of ${{Y}_{ATP}}$ given by eqn (46), we obtain


(58) \begin{eqnarray*}
{\mathrm{g\ cells\ }}\left( {{\mathrm{biomass}},{\mathrm{\ }}X} \right){\mathrm{\ }} = 0.51 \times 10.5 = 5.355\ g.
\end{eqnarray*}

Therefore, yield of biomass, *X* on glucose substrate, *S* based on actual amount of ATP consumed , the Nath-Warburg number


(59) \begin{eqnarray*}
NaWa = {{Y}_{X/S}}\ ATP = \frac{{5.355}}{{0.39 \times 180}} = \frac{{5.355}}{{70.2}} = 0.076\ g{{g}^{ - 1}}.
\end{eqnarray*}

Again, we can perform a check of the calculations: the Warburg-Nath ratio measures


(60) \begin{eqnarray*}
\frac{{\textit{Glycolysis}}}{{\textit{OXPHOS}}} = \frac{{0.255}}{{0.045}} = 5.666.
\end{eqnarray*}

Thus,


(61) \begin{eqnarray*}
{\mathrm{\ ATP\ }} &=& {\mathrm{\ lactic\ acid\ }} = \ 2 \times 5.666\\
&&\quad = 11.33\ mol{\mathrm{\ per\ mol\ glucose\ committed\ to\ OXPHOS}}.\\
\end{eqnarray*}

(62) \begin{eqnarray*}
{\mathrm{Therefore,\ total\ glucose\ consumption\ }} {=} 1 {+} 5.666 {+} 2 {=} 8.666\ mol\\
\end{eqnarray*}

and


(63) \begin{eqnarray*}
{\mathrm{ATP\ produced\ }} = 0.045 \times 11.33 = 0.51\ mol,
\end{eqnarray*}

which agrees with the value calculated earlier [eqn (55)].

## Discussion

It has been well recognized in the literature on cancer that glucose is not the sole energy source for a proliferating cell and that glutamine metabolism needs to also be considered. While it has been clear that glucose and glutamine carbons partition into biomass, lactate, and carbon dioxide, the quantitation of these contributions have not been done previously [see Figure 2 in ref. [73], and the last paragraph of their “Conclusions & Perspectives” section]. Figures 1-3 quantify these contributions based on the theory (see the “Theory” section) and stoichiometric approach (see the “Methods” section) worked out step-by-step in “Estimation of Yield Coefficients,” “Calculations of Fractions,” “Available Electron Balances,” “Calculation of the Approximate Contributions of Glucose Carbons and Glutamine Carbons to Biomass, Lactate, and CO_2_ in a Proliferating Cell,” “The Warburg Effect,” “Analysis of Warburg’s Original Data on Aerobic Glycolysis By Rat and Human Carcinoma,” and “Analysis of Warburg’s Subsequent Data Collected on Aerobic Glycolysis” sections. They show the distributions of glucose carbons as well as glutamine carbons in biomass, lactic acid, and CO_2_ (Figure 1), and also the partitioning of the total substrate carbons among these products (Figure 2). By means of the available electron balances, we have also quantified the % losses incurred in the partitioning process for the first time (Figure 3).

Modern measurements have estimated that 7%-10% of glucose uptake goes into macromolecular synthesis of cellular DNA, RNA, protein, and lipids, and ultimately into cell biomass.^73,91^ Our estimates from calculations of the biomass yield based on substrate consumed, as well as of the biomass yield based on ATP consumed [eqns (7), (48), (51), (59) and Figures 1-3] are in very good agreement with the experimental measurements. They also provide a sound theoretical basis from elemental balances that explain why these distributions into biomass, lactate, and CO_2_ are obtained. These explanations cannot be arrived at from the experimental measurements alone.

Sections “The Warburg Effect,” “Analysis of Warburg’s Original Data on Aerobic Glycolysis By Rat and Human Carcinoma,” and “Analysis of Warburg’s Subsequent Data Collected on Aerobic Glycolysis” also calculated and analyzed the biomass yields based on substrate consumed as well as the biomass yields based on ATP consumed for Warburg’s original measurements of aerobic glycolysis by rat and human carcinoma (Figure 4). Both yield coefficients, that is, ${{Y}_{X/S}}\ \textit{Glucose}$ and $NaWa = {{Y}_{X/S}}\ ATP$ satisfactorily modeled the original data on the Warburg effect (Figures 5 and 6). However, an unexpected finding of this work is that the Warburg data are explained better, with very small deviations, based on the biomass yield on ATP per unit glucose consumed, $NaWa = {{Y}_{X/S}}\ ATP$ [$n = 19;\ \textit{mean} \pm SD = 0.097 \pm 0.007$ for rat carcinoma (Figure 5), and $n = 12;\ \textit{mean} \pm SD = 0.093 \pm 0.007\ $ for human carcinoma (Figure 6)]. The biomass yield based on glucose consumed, ${{Y}_{X/S}}\ \textit{Glucose}$ fitted the data reasonably well, but revealed higher deviations [$n = 19;\ \textit{mean} \pm SD = 0.082 \pm 0.027$ for rat carcinoma (Figure 5), and $n = 12;\ \textit{mean} \pm SD = 0.098 \pm 0.028\ $ for human carcinoma (Figure 6)].

The above calculations have highlighted the superior fit of the original data on the Warburg Effect^1,3,4^ by use of ATP yields (filled green circles in Figures 5 and 6). What could be the underlying reasons for this finding? Firstly, it confirms the general notion that the energy from ATP molecules fuels biosynthetic activities and represents the best approach for estimating the energy-consuming processes of cell growth in cancer cells. Secondly, and more importantly, from the standpoint of this work, it points to a central role for energy and metabolic coupling and for intermolecular and/or intramolecular symbiosis in cancer cells. These have important biological implications, which shall now be discussed in detail.

**Figure 7.**
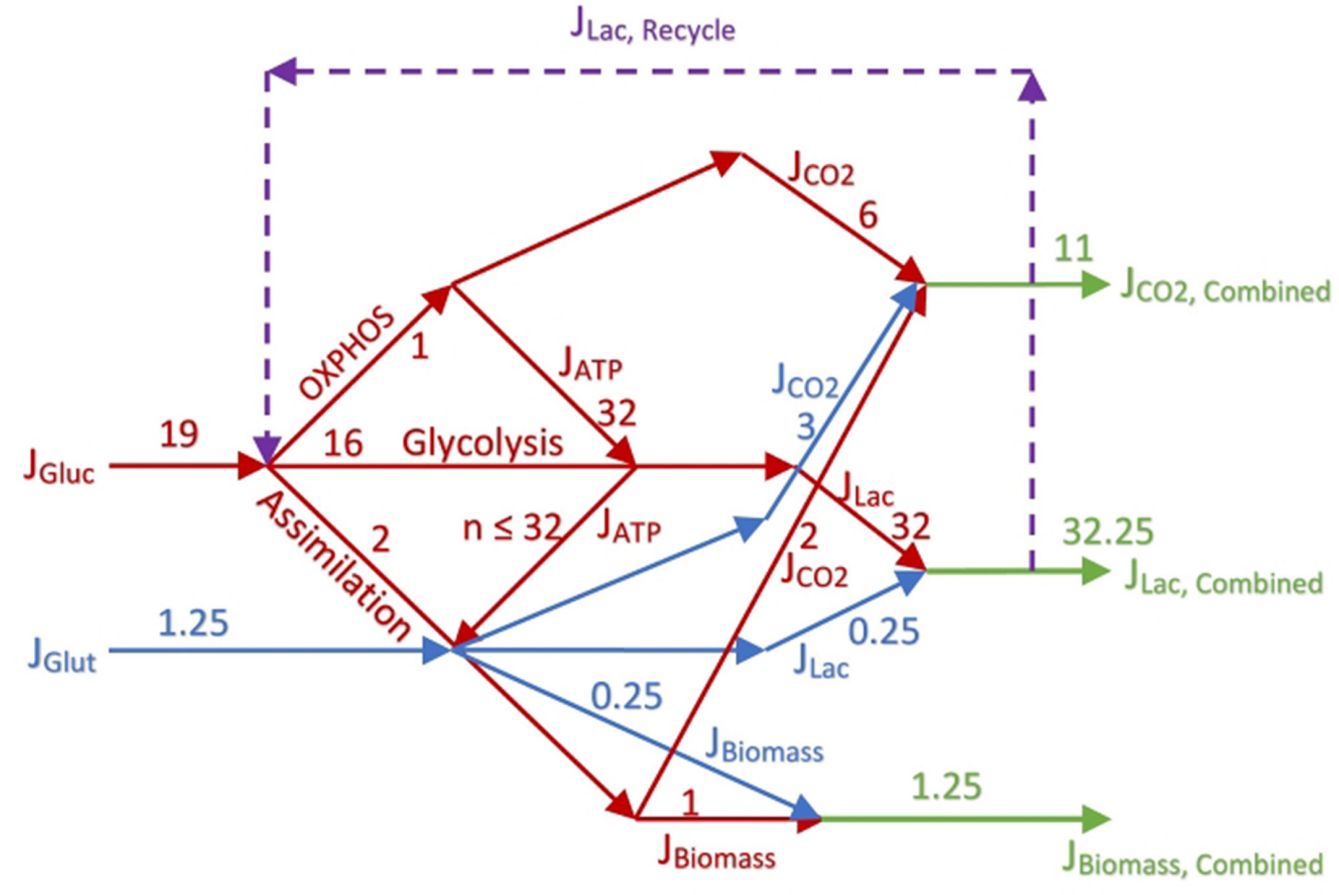
Overall mass/flux balance for an average cancer cell based on Nath's unified theory of ATP synthesis/hydrolysis.^13,41,92^ Ideally, $n = 32$ ATP molecules synthesized by OXPHOS are captured by energy coupling and used to phosphorylate 16 glucose molecules. $n < 32$ if the capture of mitochondrial ATP is not perfect. Depending on the conditions, energy demand, and tumor type, a variable percentage of the principal glycolytic product, lactate is recycled back into mitochondria and used as carbon source for making ATP by the OXPHOS process, thereby making the overall process highly efficient.

The results of “Estimation of Yield Coefficients,” “Calculations of Fractions,” “Available Electron Balances,” “Calculation of the Approximate Contributions of Glucose Carbons and Glutamine Carbons to Biomass, Lactate, and CO_2_ in a Proliferating Cell,” “The Warburg Effect,” “Analysis of Warburg’s Original Data on Aerobic Glycolysis By Rat and Human Carcinoma,” and “Analysis of Warburg’s Subsequent Data Collected on Aerobic Glycolysis” sections lead to the directed flux graph shown in Figure 7 for a proliferating cell based on Nath’s unified theory of ATP synthesis and regulation based on demand and supply^41,42^ that produces 32 ATP—after accounting for all losses on the redox and ATPase sides in mitochondria^14^—per glucose by OXPHOS (Table 1). This ATP flux is redirected into glycolysis—32 ATP phosphorylates 16 glucose molecules—and into assimilatory processes by the cell.

As shown in the “Theory” section, separate equations for biomass assimilation by each substrate, that is, glucose and glutamine, were written and analyzed by stoichiometric and available electron balances. The balance equations show that, ideally, for every 19 glucose molecules taken up by the cancer cell, 1 glucose is completely oxidized by the OXPHOS pathway. In this time, another 16 glucose molecules form lactic acid via glycolysis (Figure 7).

The results imply that the 32 ATP formed per glucose by OXPHOS initiate and regulate phosphorylation by 16 glucose molecules to form glucose-6-phosphate by the action of hexokinase 2 (HK‒II) and regenerate 32 ATP and produce 32 lactic acid molecules. If the capture of ATP generated by the process of OXPHOS at the mitochondrial contact sites by HK‒II is imperfect, or glycolytic precursors are siphoned off, then $< 32$ ATP molecules per glucose molecule are funneled into biosynthesis pathways. This implies that, in essence, the ATP produced by the OXPHOS pathway is redirected into aerobic glycolysis by the cancer cell to generate the metabolic precursors and intermediates required for biosynthesis and assimilation into biomass. These precursor molecules include glucose-6-phosphate, fructose-6-phosphate, glyceraldehyde-6-phosphate, 3-phosphoglycerate and the like. Lactic acid is a byproduct of this biosynthesis requirement. If the lactate is recycled back into the mitochondria (dashed lines in Figure 7), and used as a carbon source for the TCA cycle and OXPHOS to synthesize ATP, then we have an ideal symbiotic loop. We return to this important point at the end of this section.

In the light of the above discussion, it is incorrect to say, as is common, that proliferating cells “switch” to the less efficient/lower yield glycolytic pathway.^16,69^  **Cancer cells do not “switch.” Rather, they perform both OXPHOS and aerobic glycolysis simultaneously and concurrently by energy coupling and redirect the flux of ATP (**${{{\boldsymbol{J}}}_{{\boldsymbol{ATP}}}}$  **in**  **Figure 7****) from OXPHOS to glycolysis, and recycle the flux of the principal product, lactate (**${{{\boldsymbol{J}}}_{{\boldsymbol{Lac}}}}$**) from glycolysis back to OXPHOS (dashed lines in**  **Figure 7**). This implies that the *n* ATP molecules ($n \cong 32$) (Table 1) in OXPHOS/aerobic glycolysis are *conserved*. In other words, they are the same ATP that are usurped/hijacked from mitochondria, and used in glycolysis, which then regenerates $n \le 32$ ATP per glucose generated in OXPHOS by Nath’s two-ion theory of energy coupling and torsional mechanism of ATP synthesis.^13,14,41,60^,^63–65^,^79–82^,^92^

Hence, it is not true as often stated^55^ that in the time 1 glucose molecule synthesizes $\sim32$ ATP molecules by OXPHOS and mitochondrial respiration, an additional or extra $\sim32$ ATP molecules are generated by $\sim16$ glucose molecules via aerobic glycolysis. In fact, they are the same $\sim32$ ATP molecules produced by respiration. Of these, $n \le 32$ ATP are regenerated by glycolysis and funneled and used for assimilatory processes. This energy coupling between OXPHOS and glycolysis solves the following problems/resolves the following difficulties:

1. Regulation of distribution of glucose/lactate flux between OXPHOS and aerobic glycolysis by an energy coupling mechanism,
2. If there were to be a “switch” of pathways to glycolysis, it is difficult to conceive why resources/proteins are not re-allocated to the higher yield OXPHOS pathway. Since, no such switch exists as per our first-principles analysis, and both pathways operate simultaneously, this acute difficulty is avoided,
3. Apoptotic signals in the intrinsic mitochondrial pathway are evaded by the cancer cell by overexpressing HK‒II and anchoring them on to mitochondrial contact sites,^72^,^92^‒95
4. The well-known phenomenon of inhibition of glycolysis by glucose-6-phosphate in the cell cytosol, first described by Rose^96^ and developed in great detail by Pedersen, Ko, and coworkers.^72,97^ This type of deleterious product inhibition is avoided by anchoring HK‒II to mitochondria and by capture of ATP and by fast channeling.

The calculations in the “Results” section are based on analysis of Warburg’s experimental data^1,3,4^ applied to an averaged empirical formula of biomass composition; however, small variations in the stoichiometry of the atoms constituting the composition of biomass do not alter our results significantly. This is especially true for our key results based on the biomass yield of ATP, ${{Y}_{X/S}}\ ATP$ given by eqns (32)‒(37), (46)‒(48), (52)‒(55), (58), and (59). Further, glutamine metabolism releases NH_3_ to the cytosol and ammonia is the nitrogen source for glucose partial oxidation and assimilation into biomass. If the biomass composition of cancer cells is determined to be somewhat different, or if there exists an alternative nitrogen source, for example, HNO_3_, or glutamine itself directly, or a second product (eg, alanine) is formed, then the overall mass and electron balance equations [eqns (1)‒(6)] can be appropriately re-written. The ATP yield is given in Table 1, which is based on calculated thermodynamic efficiencies of oxidative phosphorylation.^13,14,41^ If slightly different numbers apply for a particular cell or substrate type or under different cellular conditions, then the equations can be readily re-written, and the new numbers (Figures 1-3 and 5-7) can be easily worked out. The first-principles analysis is independent of the detailed mechanism by which substrate carbons are incorporated into precursor molecules and finally into biomass using the redirected $n \cong 32$ ATP molecules per glucose.

It is indeed incredible that Warburg had made the right measurements 100 yr ago,^1^ as shown by our analysis of his data (Figures 5 and 6). The oversight then, and now^55^ was not to include/apportion ATP and glucose into biosynthetic pathways. The other analyses can be shown to make the following Generic Errors:

1. The ATP produced by the proliferating cell is the addition or sum of ATP synthesized by OXPHOS and aerobic glycolysis pathways, and not the conservation of ATP flux (Figure 7) produced by OXPHOS, which is redirected into glycolysis (symbiosis), as in Nath’s unified ATP theory,
2. Substrate glucose and/or glutamine are not apportioned into biosynthetic pathways, which ultimately use the energy of ATP molecules to accumulate biomass,
3. Warburg’s theory of 1956,^18,19^ that is, 32 yr after his original discovery of the Warburg Effect, that impaired mitochondria are a hallmark of cancer. In his words, “the respiration of all cancer cells is damaged” (ref. [18], p. 309).

As to Generic Error 3, it is not clear why Warburg proposed a primary “respiratory injury” theory, and considered it a universal metabolic hallmark of carcinogenesis,^18^ because his own original measurements of 1924^1^ showed that the rate of respiration in cancer cells is identical to that of normal cells, within the error. However, the glucose uptake was upregulated 10-15 fold in cancer cells, as already mentioned. The important 1952 work of Chance and Casto^98^ had clearly shown that cancer cells respire normally. Warburg must have been aware of this work,^98^ and other similar demonstrations. Perhaps, Warburg forgot his own experimental data on cancer cell respiration taken 32 yr earlier! In any case, the latest research has proved that, contrary to Warburg’s theory, cancer cells have functional mitochondria and reveal increased mitochondrial activity in several groups of human cancers.^21–23^,^30–34^ These studies on human tumors have employed various techniques, for example, immunochemistry and histological staining of mitochondrial OXPHOS complexes. More important from the perspective of this work, we do not require that all cancer cells in a tumor possess perfectly functional mitochondria. Given the presence of heterogeneity and metabolic symbiosis discussed above, subpopulations of functional and dysfunctional mitochondria in the tumor can co-exist, and this is sufficient for operation and regulation based on Nath’s ATP theory.

The aforementioned points 1 and 2 can also be proved. For example, a first-principles analysis on the lines of the “Results” section can be done for the (erroneous) alternative approach of Koppenol et al. (2011)^55^ that postulates a summation principle with respect to ATP produced by OXPHOS and glycolysis. As applied to Warburg’s 1926/1927 data,^3,4^ such an approach to ATP yields leads to the following numbers:


(64) \begin{eqnarray*}
{\mathrm{Glucose\ used\ for\ OXPHOS\ }} = 0.39 - 0.255 = 0.135\ mol,
\end{eqnarray*}

(65) \begin{eqnarray*}
{\mathrm{ATP\ produced\ by\ OXPHOS\ }} = 0.135 \times 32 = 4.32\ mol,
\end{eqnarray*}

(66) \begin{eqnarray*}
&&{\mathrm{Additional\ ATP\ produced\ by\ aerobic\ glycolysis\ }}\\
&&\qquad = {\mathrm{\ moles\ lactic\ acid\ }} = 0.255 \times 2 = 0.51\ mol,
\end{eqnarray*}

(67) \begin{eqnarray*}
{\mathrm{Total\ ATP\ produced\ }} = 4.32 + 0.51 = 4.83\ mol,
\end{eqnarray*}

(68) \begin{eqnarray*}
&&{\mathrm{\% \ excess\ ATP\ generated\ by\ cancer\ cells\ over\ normal\ cells\ }}\\
&&\qquad = \frac{{0.51}}{{4.83}} \times 100 = 10.56\%,
\end{eqnarray*}

in agreement with the calculations of Koppenol et al. (p. 329, ref. [55]). However,


(69) \begin{eqnarray*}
{{Y}_{X/S}}\ ATP = \frac{{4.83 \times 10.5}}{{0.39 \times 180}} = \frac{{50.715}}{{70.2}} = 0.722\ g{{g}^{ - 1}}{\mathrm{\ or\ }}72.2{\mathrm{\% }},
\end{eqnarray*}

which is an order of magnitude off from the values of $7\% - 10\% $ by experiment,^73^ and also from the value calculated by our approach [eqn (59)]. Further, such a summation approach does not predict Warburg’s data on aerobic glycolysis accurately (Figures 5 and 6). These differences arose due to Generic Error 1 listed above.

The error is not corrected even if we assume imperfect capture of ATP and use the actual value by experiment of 11.33 ATP per glucose [eqns (60) and (61)]. This gives the numbers as follows:


(70) \begin{eqnarray*}
&&{\mathrm{ATP\ produced\ by\ OXPHOS\ with\ imperfect\ capture\ }} = 0.135\\
&&\qquad \times 11.33 = 1.53\ mol,
\end{eqnarray*}

(71) \begin{eqnarray*}
{\mathrm{Total\ ATP\ }} = 1.53 + 0.51 = 2.04\ mol,
\end{eqnarray*}

(72) \begin{eqnarray*}
{{Y}_{X/S}}\ ATP = \frac{{2.04 \times 10.5}}{{0.39 \times 180}} = \frac{{21.42}}{{70.2}} = 0.305\ g{{g}^{ - 1}},
\end{eqnarray*}

which again does not explain the data in Figures 5 and 6 or the data summarized in reviews.^73^

Similar results are obtained for Warburg’s original data of 1924 on aerobic glycolysis.^1^ With perfect capture, the summation approach^55^ yields ${{Y}_{X/S}}\ ATP = 0.41\ g{{g}^{ - 1}}$, and ${{Y}_{X/S}}\ ATP = 0.293\ g{{g}^{ - 1}}$ with less-than-perfect ATP capture from mitochondrial contact sites/junctions, which is again very far off from the actual values (Figures 5 and 6) and from the estimates made by eqns (48) and (51).

Other analyses also face the generic difficulties listed previously or are incomplete descriptions. For example, classical work on ^14^C labeling in mouse L-M strain fibroblasts grown on 11 m m glucose estimated that 55% of ^14^C substrate is converted to ^14^CO_2_.^99^ From these studies, it was concluded that this contributes 35% of the ATP requirement for these cells.^73,99^ However, the analysis uses the complete oxidation reaction [eqn (1)], and not the full set of reactions [eqns (3)‒(6)]. Thus, 55% of 6 CO_2_ [eqn (1)] gives 3.3 CO_2_, and therefore its ATP contribution on the basis of eqn (1) works out to be $\frac{{3.3}}{{6 + 3.3}} \times 100 = 35\% $. Hence, biomass accumulation was not considered in these calculations, thereby effectively making Generic Error 2.

In summary, the correct NaWa numbers/yields are obtained [ie, with ${{Y}_{X/S}}\ ATP$ given by eqns (48) and (59) for Warburg’s data on rat carcinoma^1,3,4^ (Figure 5), and similarly for human carcinoma (Figure 6)] ONLY IF the flux of ATP, ${{J}_{ATP}}$ is conserved and, ideally, redistributed from OXPHOS to glycolysis (Figure 7), and thereafter into assimilatory processes, as postulated by Nath’s unified theory of ATP synthesis/hydrolysis. A nonconservative (additive) summation approach to the problem of ATP utilization for cell growth and proliferation does not give correct results. We conclude that a conservation principle with respect to ATP accurately predicts yield coefficients for energy metabolism by cancer cells.

Equations (48) and (59) estimate the biomass yield ${{Y}_{X/S}}\ ATP$ based on the actual amount of ATP consumed determined experimentally and used for producing cell biomass. These actual yield coefficients based on ATP consumed have been shown to accurately predict the Warburg Effect data on aerobic glycolysis on rat and human carcinoma (Figures 5 and 6). Since good experimental data are available, one could obtain the exact operative values of ${{Y}_{X/S}}\ ATP$, that is, of actual yield coefficients based on ATP consumed. However, even the ideal ATP yield per glucose would have approximately (though not perfectly) modeled the data on the Warburg Effect shown in Figures 5 and 6. Thus, for the data analyzed in the “Analysis of Warburg’s Original Data on Aerobic Glycolysis By Rat and Human Carcinoma” section, we estimate, the ideal Nath-Warburg number


(73) \begin{eqnarray*}
NaWa,ideal &=& {{Y}_{X/S}}\ ATP,\\ \textit{ideal} &=& \frac{{0.98 \times 10.5}}{{\left( {\frac{{0.98}}{{32}} \times 3 + 0.49} \right) \times 180}} = \frac{{10.29}}{{104.7375}}\\ &=& 0.098\ g{{g}^{ - 1}},
\end{eqnarray*}

and, similarly for the data analyzed in the “Analysis of Warburg’s Subsequent Data Collected on Aerobic Glycolysis” section,


(74) \begin{eqnarray*}
NaWa,ideal &=& {{Y}_{X/S}}\ ATP,\ \textit{ideal}\\ &=& \frac{{0.51 \times 10.5}}{{\left( {\frac{{0.51}}{{32}} \times 3 + 0.255} \right) \times 180}} = \frac{{5.355}}{{54.506}}\\ &=& 0.098\ g{{g}^{ - 1}}.
\end{eqnarray*}

These should be compared with $NaWa = {{Y}_{X/S}}\ ATP,\ \textit{actual}$ of $0.097$  $g{{g}^{ - 1}}$ [eqn (48), Figure 5] and $0.076$  $g{{g}^{ - 1}}$ [eqn (59)], respectively.

## Concluding Remarks and Biological Implications

What is the relationship of this work’s first-principles analysis of the Warburg Effect with modern biological studies? What are the major results, conclusions, and biological implications of the work from the perspective of ATP metabolism, OXPHOS bioenergetics, and the tumor microenvironment?

1. Metabolic heterogeneity within human tumors is a well-known phenomenon. The tumor core is hypoxic and glycolytic, while cells in the tumor surface are oxygenated and rely on OXPHOS for ATP synthesis by the F_0_F_1_-ATP synthase. The metabolic interaction and coupling between stromal cells and cancer cells, or symbiosis between fibroblasts and cancer cells, in astrocytes and neurons, and in intervertebral disc cells has been well-documented.^28,34,50,66^ Thus, supporting stromal cells exhibit a glycolytic phenotype and interact by catabolite transfer with adjacent cancer cells that predominantly utilize OXPHOS for ATP production. Such compartmentalization among cells has also been called the “Reverse Warburg Effect.”^23^,^32–40^ The transfer of catabolites required for such metabolic coupling and symbiosis between cells includes monocarboxylate anions such as lactate. Thus, lactate produced by glycolytic cells in the hypoxic tumor core is translocated and used to carry out OXPHOS and ATP synthesis by cells in the more oxygenated outer layer of the tumor. The present analysis applies not only to such intercellular energy coupling, but also in the context of intracellular coupling (Figure 7).
2. Figure 7 shows that the product ATP synthesized by the F_0_F_1_-ATP synthase^60^,^79–82^ by the process of OXPHOS in mitochondria^13,14,41,42^ can be redistributed and harnessed for substrate level phosphorylation in the cytosol of cancer cells. Part of the product lactate of the glycolytic cascade can be recycled back into mitochondria, converted therein to pyruvate by the action of lactate dehydrogenase, which can enter the TCA cycle, and be metabolized via OXPHOS (Figure 7). Thirty-two ATP can be synthesized per 2 lactate molecules (Table 1), and hence, this energy-rich molecule and carbon source is not wasted. Recent experimental works support such utilization of lactate by the mitochondrial electron transport chain.^48,50,51^ Such energy coupling between cells—and also within cells—would maximize cell proliferation and growth (this work).
3. It ought to be emphasized that the 32 ATP molecules synthesized per glucose (or per 2 lactic acid molecules) (Table 1) represents the final net actual ATP production, that is, after accounting for all losses, such as membrane leaks, respiratory slips, active transport losses^16^ as per Nath’s two-ion theory of energy coupling^26,27,41,63^^‒^^65,81,82^ and torsional mechanism of ATP synthesis.^60,83,92^ This differs from the standard treatments where $\sim$32 ATP per glucose is the ideal, mechanistic value that would require further downgrades to account for losses, as correctly interpreted by Levy and coworker in their comprehensive ATP energy audit for the brain.^100^
4. The emerging consensus on lactic acid as an energy-rich metabolite makes “a plea to consider the key role of lactate anion, as opposed to hydrogen ion in cancer cell metabolism.”^53^ The situation is very similar to the central role of the succinate anion, separate from that of the H^+^ ion postulated by Nath’s two-ion theory of energy coupling and ATP synthesis in OXPHOS in cell life.^60,63,79,80,92^ This concept has been generalized,^13,82^ quantified,^14,81^ and considered to be of vital importance for energy coupling, homeostasis, and regulation.^41,42^ For instance, the V-type vacuolar ATPase is responsible for acidification of organelles. However, such acidification of a large space cannot be achieved by translocating a proton alone, from basic physical chemistry concepts, but requires also that an anion such as succinate (or counter-cation such as K^+^) be *co-transported* by the enzyme.^13^
5. The important question of why the grade of malignancy differs among cancer cell types has been posed.^28,29^ However, neither genomic studies nor metabolic studies of cancer have fully answered this question. A single hypoxia or glycolysis score, or the enhanced glycolytic rate of the Warburg Effect discussed in this work do not constitute a satisfactory index for categorizing malignancy in terms of cell proliferation and metastasis. Based on the first-principles analysis in the “Results” section of this work, and its detailed discussion in the “Discussion” section, a *hybrid* index or score that takes into consideration both glycolysis and OXPHOS rates and thereby models metabolic coupling/symbiosis in cancer cells appears to be necessary.
6. In the context of this work, a superior index that can better correlate variable malignancies in cancers is given by the equation,
  (75) \begin{eqnarray*}
\textit{Degree}\ of\ \textit{malignancy}\ \propto NaWa = {{Y}_{X/S}}\ ATP.
\end{eqnarray*}
  The value of the actual yield coefficient based on ATP in eqn (75) varies between $0$ (no proliferation or energy coupling) to the ideal Nath-Warburg number ${{Y}_{X/S}}\ ATP,\ \textit{ideal}$ (maximum energy coupling/malignancy) [$= 0.098\ g{{g}^{ - 1}}$ based on Nath’s unified theory of ATP synthesis/hydrolysis and regulation (Table 1) and eqns (73) and (74)]. Rat and human carcinoma tissues analyzed here lie somewhere in between these 2 limits [eqns (48) and (59) and Figures 5 and 6].
7. Equation (75) provides a *composite* index from the perspective of ATP metabolism in cancer cells that takes into account both glycolysis and OXPHOS. It is also a measure of the ratio of substrate utilized for aerobic glycolysis versus substrate utilized for OXPHOS in the heterogeneous cancer cell populations [eqns (43) and (60)]. It is advocated as an interesting index for consideration and further testing by workers in the field of cancer research. A potential approach to test this suggestion is by examination of OXPHOS and glycolytic activities across pan-cancer data, for example, by use of The Cancer Genome Atlas of the NIH.^101^ The authors would like to acknowledge the reviewers for pointing this approach that is pregnant with possibilities for future work.
8. The fundamental concepts of stoichiometry and biomass assimilation, available electron balance, and ATP yield are very general and should hold across cancer cell types and conditions. The fundamental understanding of the entire process of bidirectional coupling between aerobic glycolysis and OXPHOS gained using a new perspective from ATP mechanism should help in catalyzing the development of more efficient metabolism-targeting anticancer drugs.

## Data Availability

The data underlying this article will be shared on reasonable request to the corresponding authors.
